# Talking the same language on patient empowerment: Development and content validation of a taxonomy of self‐management interventions for chronic conditions

**DOI:** 10.1111/hex.13303

**Published:** 2021-07-12

**Authors:** Carola Orrego, Marta Ballester, Monique Heymans, Estela Camus, Oliver Groene, Ena Niño de Guzman, Hector Pardo‐Hernandez, Rosa Sunol, COMPAR‐EU Group

**Affiliations:** ^1^ Avedis Donabedian Research Institute (FAD) Barcelona Spain; ^2^ Universitat Autònoma de Barcelona (UAB) Barcelona Spain; ^3^ Red de investigación en servicios de salud en enfermedades crónicas (REDISSEC); ^4^ Netherlands Institute for Health Services Research (NIVEL); ^5^ OPTIMEDIS; ^6^ London School of Hygiene and Tropical Medicine London UK; ^7^ Iberoamerican Cochrane Centre ‐ Biomedical Research Institute Sant Pau (IIB Sant Pau) Barcelona Spain; ^8^ CIBER de Epidemiología y Salud Pública (CIBERESP) Barcelona Spain

**Keywords:** chronic diseases, comparative effectiveness, intervention reporting, patient empowerment, patient‐centred care, self‐management

## Abstract

**Context:**

The literature on self‐management interventions (SMIs) is growing exponentially, but it is characterized by heterogeneous reporting that limits comparability across studies and interventions. Building an SMI taxonomy is the first step towards creating a common language for stakeholders to drive research in this area and promote patient self‐management and empowerment.

**Objective:**

To develop and validate the content of a comprehensive taxonomy of SMIs for long‐term conditions that will help identify key characteristics and facilitate design, reporting and comparisons of SMIs.

**Methods:**

We employed a mixed‐methods approach incorporating a literature review, an iterative consultation process and mapping of key domains, concepts and elements to develop an initial SMI taxonomy that was subsequently reviewed in a two‐round online Delphi survey with a purposive sample of international experts.

**Results:**

The final SMI taxonomy has 132 components classified into four domains: intervention characteristics, expected patient/caregiver self‐management behaviours, outcomes for measuring SMIs and target population characteristics. The two‐round Delphi exercise involving 27 international experts demonstrated overall high agreement with the proposed items, with a mean score (on a scale of 1‐9) per component of 8.0 (range 6.1‐8.8) in round 1 and 8.1 (range 7.0‐8.9) in round 2.

**Conclusions:**

The SMI taxonomy contributes to building a common framework for the patient self‐management field and can help implement and improve patient empowerment and facilitate comparative effectiveness research of SMIs.

Patient or public contribution.

Patients’ representatives contributed as experts in the Delphi process and as partners of the consortium.

## 1 BACKGROUND

Continuous progress towards patient‐centred care has supported the emergence of a new paradigm in which patients are no longer passive recipients of care, but increasingly take an active role in the co‐production of their health.^1^ This shift has been accompanied by an increasing interest in self‐management interventions (SMIs), as reflected by the significant growth in scientific output in this field. Published between 2010 and 2015 alone were over 257 systematic reviews examining randomized control trials (RCTs) on SMIs for seven chronic conditions.^2^

Self‐management of a health condition has been defined as ‘what individuals, families and communities do with the intention to promote, maintain, or restore health and to cope with illness and disability with or without the support of health professionals....’.^3^ In practice, self‐management of a long‐term health condition requires that a person has self‐efficacy (confidence in their ability to cope with the disease^4^ ), which entails acquiring the skills needed to monitor symptoms and clinical markers, understand their implications and adjust treatment and behaviours accordingly (eg lifestyle, treatment adherence/compliance, work and other daily activities). Drawing on this definition, SMIs can be characterized as supportive interventions that health‐care staff, peers or laypersons systematically provide to increase patients’ skills and confidence in their ability to manage long‐term conditions. SMIs aim to equip patients (and, where appropriate, informal caregivers) in such a way that they can actively participate in the management of conditions.^5^

With the increasing attention being paid to patient self‐management, questions have emerged about the extent to which SMIs are effective. The enormous number of systematic reviews and meta‐analyses in this field has aimed to provide an unambiguous answer about the effectiveness of SMIs, but has repeatedly highlighted the issue of great heterogeneity in SMIs. The way SMIs are defined ultimately determines study selection for these systematic reviews and meta‐analyses from which conclusions are drawn. Many studies give merely a conceptual or general definition of SMIs, or no definition at all. The heterogeneity is further exacerbated by diverse and inaccurate reporting of intervention components, delivery methods and outcomes. Variations in terminology and definitions limit the possibility and reliability of comparisons, complicating the translation of research into practice. They also impede the correct identification of successful intervention components, making it difficult to describe how and under what conditions an intervention is optimally implemented.

One way to address this problem and facilitate a better definition of SMIs is by establishing a taxonomy. Taxonomies are formal systems for classifying multifaceted complex phenomena into sets of common conceptual domains and dimensions.^6^ A taxonomy with a comprehensive list of SMI components can facilitate primary research, as intervention developers would be able to draw on a wider range of important components than is likely to be considered without such a list. Specifying intervention and control conditions using a taxonomy would increase accurate replication of SMIs found to be efficacious in RCTs, and would be useful for assessing the fidelity of SMI implementation. For researchers performing systematic reviews, a taxonomy would provide a reliable method for extracting and coding information about SMI content, while reviewers could identify and synthesize discrete, replicable, potentially active ingredients associated with effectiveness.

Specifying SMI content would help to maximize the scientific as well as practical benefits of research investment in the development and evaluation of complex interventions. This would lead to a transparent selection process for SMIs being studied or evaluated in research reports, leading to more correct conclusions about the effectiveness of SMIs.

Although significant advances have been made in defining SMI taxonomies in recent years, ^7^, ^8^, ^9^, ^10^, ^11^ no existing taxonomy has been developed on the basis of a confirmatory process by experts external to the research team. Furthermore, most focus only on self‐management support or behavioural change techniques, with little attention given to other components that are equally relevant to SMI design, implementation and reporting. As a result, there is no wide‐ranging classification of SMIs that can inform policy or research in the field by allowing comparisons across studies or incorporating implementation considerations.

The aim of this study, conducted within the European‐funded COMPAR‐EU project on SMIs,^12^ was to develop and validate the content of a comprehensive consensus‐based taxonomy of SMIs addressing the needs of patients living with chronic conditions. In addition, this study aimed to build a common framework to facilitate the evaluation and comparison of the effectiveness and cost‐effectiveness of SMIs, so as to promote reproducible and comparable research in this field.

## 2 METHODS

### 2.1 Study design and setting

The taxonomy was developed using a mixed‐methods approach involving (1) a literature review; (2) content analysis to map selected data sources, identify key domains, concepts, and elements and develop a preliminary taxonomy; and (3) content validation by a two‐round Delphi survey of international experts.

### 2.2 Qualitative review of the literature

#### 2.2.1 Data sources and searches

We performed a literature review of studies as follows: (1) we desk‐reviewed studies identified in a previous European project on promoting self‐management of chronic diseases in Europe (PRO‐STEP)^2^ which included an overview of 257 systematic reviews; (2) we conducted electronic searches in PubMed using the keywords ‘self‐management’, ‘self‐care’, ‘taxonomy’, ‘classification system’ and ‘classification’, combined as follows: ‘self‐management AND taxonomy’, ‘self‐care AND taxonomy’; ‘self‐management AND classification system’; ‘self‐care AND classification system’; ‘self‐management AND classification’; and ‘self‐care AND classification’; and (3) we hand‐searched reference lists of relevant systematic reviews.

Inclusion criteria were publications focused on self‐management of chronic conditions that included a definition of self‐management and that described a classification system or a set of structured attributes for characterizing SMIs.

#### 2.2.2 Data analysis and taxonomy building

A qualitative approach was used to build the draft version of the SMI taxonomy^13^ that included the following steps.

##### *Mapping* *selected data* *sources*

Content analysis techniques were used to analyse the selected literature^6^ and create a preliminary map of SMI components.^13^ A deductive approach was used to create components, building on previous work done in PRO‐STEP.^2^

##### *Identifying* *and naming* *concepts*

The preliminary mapping strategy and taxonomy structure were proposed by the team of researchers. A short definition (including some examples) was created for each component of the taxonomy, including the naming of concepts.

##### *Deconstructing* *and categorizing* *concepts*

The components were organized and categorized according to their features using an iterative process and team discussions. The key variables for discussion were relevance, clarity, identification of missing components, an understanding of all components included their interrelatedness and selection of clear labels. ﻿

##### *Integrating* *concepts and final* *synthesis*

A first draft of the taxonomy was agreed on by the researchers, resulting in a set of concepts organized in three levels of aggregation (domains, subdomains and elements), short definitions for the components and an illustrative conceptual map of the taxonomy.

### 2.3 Validation of the proposed taxonomy

The content of the proposed taxonomy was validated using a Delphi consensus survey, conducted as described below.

### 2.4 Selection of participants

Purposive sampling was used to select experts in SMIs and/or taxonomies from among the following groups: (1) authors of existing taxonomies; (2) individuals with scientific expertise in SMIs and authors of publications in this field (including authors of the systematic reviews identified in PRO‐STEP^2^ ); (3) other recommended experts on self‐management, shared decision making and patient empowerment; and (4) patient representatives. The candidate participants received an e‐mail describing the objectives and details of the study and inviting them to participate in the Delphi consensus survey.

### 2.5 Delphi consensus survey round 1

Individuals who agreed to participate received a link to the online Delphi survey tool along with a personalized username and password. On entering the survey tool, participants were presented with an interactive mind map of the draft SMI taxonomy (as created by the researchers in the first stage of the project) that described the overall structure and components of a draft taxonomy. Participants were then asked to score each component, in terms of its importance for inclusion in an SMI taxonomy, on a scale of 1 (not important) to 9 (extremely important). They could also indicate ‘I don't know’ or provide further suggestions in a free‐text field.

Mean, standard deviation (SD) and median scores were calculated to classify components as follows: components to be eliminated (those with a mean score of less than 7) and components to remain (those with a mean score of 7 or higher). Where appropriate, component labels and descriptions were modified, and components were merged or split based on participants’ suggestions. Components were also added if one or more experts provided adequate justification. Conflicting suggestions from participants were discussed and resolved by members of the research team.

### 2.6 Delphi consensus survey round 2

For each component from round 1, participants were presented with the mean (SD) scores and median scores for the overall group. Any changes that had been made to the draft taxonomy were also shown graphically. Participants also received descriptions as refined using qualitative inputs from round 1. Participants were then asked to score each component again in round 2. They also had the option to provide further suggestions. The same scoring rules as for round 1 were applied.

### 2.7 Approval of the final taxonomy

On observing the high agreement found among experts and the saturation of qualitative information after round 2 of the Delphi consensus survey, consensus was considered to have been reached. Two researchers made the final changes based on second‐round results and presented the final taxonomy to the research team. Final minor editing and wording improvements were made based on inputs from the research team.

### 2.8 Ethical considerations

Ethical approval for this study was obtained. All procedures were carried out in accordance with the principles of the Declaration of Helsinki and subsequent amendments. All participants in the online Delphi survey received detailed information about the project and provided informed consent. The former was a requirement to access the platform.

## 3 RESULTS

### 3.1 Qualitative literature review and taxonomy building

The literature review retrieved 49 publications meeting the inclusion criteria and containing potentially useful classification systems of component attributes: 38 systematic reviews,^14^, ^15^, ^16^, ^17^, ^18^, ^19^, ^20^, ^21^, ^22^, ^23^, ^24^, ^25^, ^26^, ^27^, ^28^, ^29^, ^30^, ^31^, ^32^, ^33^, ^34^, ^35^, ^36^, ^37^, ^38^, ^39^, ^40^, ^41^, ^42^, ^43^, ^44^, ^45^, ^46^, ^47^, ^48^, ^49^, ^50^, ^51^ six publications identified through snowballing techniques^52^, ^53^, ^54^, ^55^, ^56^, ^57^ and five taxonomies that partially covered SMI components.^7^, ^8^, ^9^, ^10^, ^11^ Using the methods described above, a first draft and conceptual mapping of the taxonomy were created, containing 127 components structured hierarchically into three levels: domains (n = 4), subdomains (n = 19) and elements (n = 104). The initial draft taxonomy was composed as follows:

1. Intervention characteristics, with subdomains as follows: self‐management support technique; support delivery method; provider type; location; and recipient. These subdomains may independently or interdependently interact to increase self‐management skills.
2. Expected patient/caregiver self‐management behaviours, defined as the self‐management decisions and behaviours engaged in by patients with long‐term conditions (or their caregivers) that affect health, that is, changes they are expected to make in order to manage their disease better in accordance with their needs, for example specific health problems, contextual factors and personal preferences. The subdomains were as follows: lifestyle‐related behaviours; clinical management; psychological management; social management; and working with health‐care or social care providers.
3. Outcomes for measuring SMIs, reflecting measures of SMI effects and featuring the following subdomains: basic empowerment components; level of adherence to expected self‐management behaviours; clinical outcomes; patient/caregiver quality of life (where caregiver is understood to be an informal caregiver, ie a friend or family member); care perceptions/satisfaction; health‐care use; and costs.
4. Target population, broken down into subdomains as defined by intervention recipients (patients/caregivers), disease‐related characteristics, and socioeconomic and demographic characteristics.

### 3.2 Delphi survey

Thirty‐three experts (14% of those contacted, 85% of respondents to the invitation) completed the online Delphi survey round 1, and 27 of those 33 experts (81%) completed round 2. Their characteristics are summarized in Table 1. The background of the group was balanced, including as it did, experts from clinical disciplines, epidemiology and public health. Most participants that completed both rounds were researchers (81%, n = 22), while the remainder were health‐care providers (18.5%, n = 5), patient representatives (11%, n = 3) and policymakers (7%, n = 2). Overall, the participants were from nine countries and had a mean of 9.64 years’ experience in the self‐management field.

**Table 1:** Delphi participant characteristics and response rates

	Round 1	Round 2
Participation		
Total number of experts invited	231	33
Did not respond	192	2
Started but did not finish	6	5
Declined to participate	–	1
Total number of experts who completed survey	33	27
Response rate (%)	14.28	81.81
Expert characteristics		
Country	n %	n %
United Kingdom	10 (30.3)	9 (33.3)
United States of America	5 (15.2)	2 (7.4)
Australia	4 (12.1)	4 (14.8)
Spain	4 (12.1)	3 (11.1)
Canada	3 (9.1)	3 (11.1)
Ireland	3 (9.1)	3 (11.1)
Belgium	2 (6.1)	1 (3.7)
Germany	1 (3.0)	1 (3.7)
Norway	1 (3.0)	1 (3.7)
Background^a^	n (%)	n (%)
Epidemiology/public health	12 (36.4)	10 (37.0)
Medicine	7 (21.2)	6 (22.2)
Nursing	7 (21.2)	5 (18.5)
Psychology	5 (15.2)	4 (14.8)
Sociology	3 (9.1)	3 (11.1)
Diet/nutrition	2 (6.1)	2 (7.4)
Pharmacy	1 (3.0	1 (3.7)
Others (social work, sociology, health service research)	8 (24.2)	7 (25.9)
Position^b^	n (%)	n (%)
Researcher	28 (84.8)	22 (81.5)
Health‐care provider	6 (18.2)	5 (18.5)
Academic	4 (12.1)	3 (11.1)
Patient representative	3 (9.1)	3 (11.1)
Policymaker	2 (6.1)	2 (7.4)

^a^ Some participants had backgrounds in more than a single field.

^b^ Some participants had more than a single position.

### 3.3 General and specific levels of agreement

The overall level of agreement was high throughout the Delphi exercise, with a mean score (on a scale of 1‐9) per component of 8.0 (range 6.1‐8.8) in round 1 and 8.1 (range 7.0‐8.9) in round 2.

Three of the four top‐level domains scored highest in round 2, namely, intervention characteristics (mean 8.93, SD 0.26), outcomes for measuring SMIs (mean 8.93, SD 0.26) and expected patient/caregiver self‐management behaviours (mean 8.85, SD 0.45). Of the six subdomains with the highest scores (≥8.5), self‐management support techniques (mean 8.67, SD 0.47) and support delivery methods (mean 8.67, SD 0.61) were the highest‐ranked subdomains, both belonging to the intervention characteristics domain. Finally, of the 14 elements with the highest scores (≥8.5), the two highest‐ranked were patients (mean 8.81, SD 0.62) and community‐based care (mean 8.76, SD 0.5), from the target population domain and the intervention characteristics domain, respectively.

In round 2, support delivery method elements obtained the lowest scores (<7.5): specific devices (mean 7.08, SD 1.81), layperson, service (mean 7.14 each, SD 2 and SD 1.55, respectively) and specific population (mean 7.21, SD 1.61).

### 3.4 Review of changes

A significant proportion of the changes made to the draft taxonomy as a result of the two‐round Delphi survey were modifications to existing components. In total, 144 changes were made (81 after round 1 and 63 after round 1). Table 2 summarizes the refinement process and tracks changes resulting after each Delphi round. Most of the changes (71.5%, n = 103) were changes to labels to clarify meaning (see Table S2).

**Table 2:** Taxonomy refinement process and changes tracked after expert consultation

Round	Type of change	Domains	Subdomains	Elements
Round 1	Included	4	26	107
	Eliminated	0	1	1
	Modified	0	6	4
Round 2	Included	4	25	103
	Eliminated	0	1	4
	Modified	0	6	12
	Merged	0	3	1
	Added	0	0	5
Final taxonomy	Included	4	25	103

Merging was proposed just once, for two elements (in round 1), and resulted in the combination of coaching with motivational interviewing as a single element in the self‐management support technique subdomain. Additions (only proposed in round 2) were five elements as follows: specific population in the recipient subdomain, health‐care assistant in the provider type subdomain, healthy sleep habits in the lifestyle‐related behaviours subdomain, direct non‐medical costs in the costs subdomain and digital literacy in the socioeconomic/demographic characteristics subdomain. Eliminations (only suggested in round 1) included educational and/or training sessions from the encounter type subdomain, mail and physical educational materials from the support delivery mode subdomain and emergency care from the locations subdomain. Finally, based on suggestions received in round 2, social/sexual functioning in the patient/informal caregivers’ quality of life subdomain was split into social functioning and sexual functioning. Input from experts throughout the Delphi exercise was also used to improve definitions of elements and examples (see Table S3).

### 3.5 Definitive self‐management intervention taxonomy

**Figure 1:**
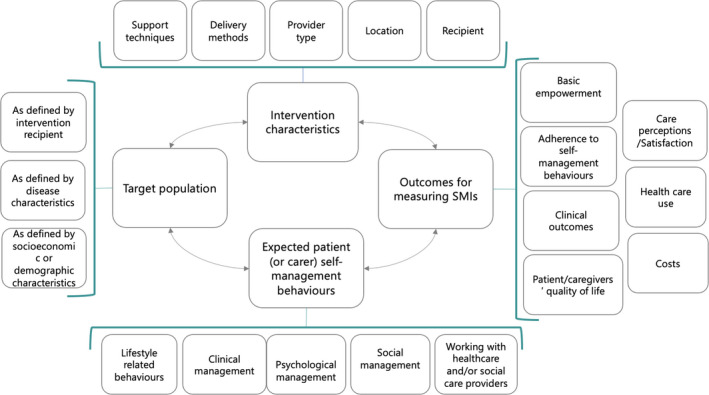
Main components and conceptual relationships of the definitive self‐management intervention taxonomy

Tables 3 and 4 present the definitive taxonomy of SMIs, including 132 components organized in four domains (the same as in the draft taxonomy), now rearranged in 25 subdomains and 103 elements. The main levels of the taxonomy are shown in Figure 1.

**Table 3:** Final self‐management intervention taxonomy (domains 1 and 2)

Subdomain	Elements
Domain 1: Self‐management intervention characteristics	
1.1 Support technique	Sharing information, skill training, stress and/or emotional management, shared decision‐making, goal setting and action planning, problem‐solving skill enhancement, self‐monitoring training and feedback, using prompts and reminders, encouraging the use of services, providing equipment, social support, coaching and motivational interviewing
1.2 Delivery method
*Subdivided into three subdomains:*	
1.2.1 Encounter type	Clinical visit, support session and self‐guided intervention
1.2.2 Support delivery mode
Subdivided into 2 subdomains:
1.2.2.1. Face‐to‐face intervention
1.2.2.2. Distance/remote intervention	Phone calls, smartphone, Internet, specific devices
1.2.3 Time of communication	Synchronous and asynchronous
1.3 Recipient	Individual, group and specific population
1.4 Provider type	Physician, nurse, pharmacist, physiotherapist, occupational therapist, social worker, psychologist, dietician/nutritionist, health‐care assistant, peer, layperson and service
1.5 Location	Hospital (inpatient care), long‐term centre/nursing home care, community‐based care, home care, primary care, outpatient setting, workplace
Domain 2: Expected patient/caregiver self‐management behaviours	
2.1 Lifestyle‐related	Eating behaviours, physical activity/exercise, smoking cessation or reduction, cessation or reduction of the consumption of alcohol or other harmful substances and healthy sleep habits
2.2 Clinical management	Condition‐specific behaviours, self‐monitoring, medication use and adherence, early recognition of symptoms, asking for professional help or emergency care when needed, device management and physical management
2.3 Psychological management	Handling/managing emotions
2.4 Social management	Fitting in at work, social roles and being able to work
2.5 Working with health‐care/social care providers	Communicating with health‐care and/or social care providers

**Table 4:** Final self‐management intervention taxonomy (domains 3 and 4)

Subdomain	Elements
Domain 3: Outcomes for measuring SMIs	
3.1 Basic empowerment	Level of knowledge,‐ level of health literacy, level of skill acquisition, level of self‐efficacy and level of patient activation
3.2 Adherence to self‐management behaviours	Lifestyle‐related behaviours, clinical self‐management behaviours, psychological self‐management behaviours, social self‐management behaviours, interactions and communication with health‐care/social care providers
3.3 Clinical outcomes	Disease progression (clinical markers, symptoms), complications, adverse events and mortality
3.4 Patient/caregiver quality of life	Overall quality of life, physical functioning, psychological and emotional functioning, social functioning, sexual functioning and burden of treatment
3.5 Care perceptions/satisfaction	Overall satisfaction with self‐management interventions, perceptions of being well and sufficiently informed (quality of information provision), perceptions of patient‐provider relationship and personalized care
3.6 Health‐care use	Type and number of visits, hospital admissions and readmissions and emergency care
3.7 Costs	Health‐care costs for patients, health‐care costs, direct non‐medical costs and societal costs
Domain 4: Target population	
4.1 As defined by intervention recipient	Patients, informal caregivers or family caregivers
4.2 As defined by disease‐related characteristics	Time since diagnosis, disease severity, comorbidity and multimorbidity
4.3 As defined by socioeconomic/ demographic characteristics	Socioeconomic status, cultural group, health literacy, digital literacy, biological sex or gender, age and living situation

The intervention characteristic domain (domain 1 in Table 3) has five main subdomains. The self‐management support technique subdomain, considered one of the most important SMI components, features a range of strategies, including basic techniques like sharing information and more complex, specialized approaches, such as coaching and motivational interviewing. Support delivery methods are subdivided into type of encounter, mode of delivery (remote and/or face to face) and time of communication, which may be synchronous or asynchronous. The recipient subdomain distinguishes between interventions targeting individuals, groups and specific populations. The provider type subdomain covers a long list of potential providers ranging from health‐care professionals to peers and laypersons. Finally, the location subdomain includes health‐care centres at different levels, patient's homes, the local community and workplaces.

The expected patient/caregiver self‐management behaviour domain (domain 2 in Table 3) is composed of the five subdomains of lifestyle‐related behaviours, clinical management (including condition‐related and self‐monitoring behaviours and other behaviours such as medication adherence), psychological management (handling and managing emotions), social management and working with health‐care or social care providers.

The outcomes for measuring SMI domain (domain 3 in Table 4) cover seven subdomains: basic empowerment components (eg level of knowledge or health literacy), level of adherence to expected self‐management behaviours, clinical outcomes (eg disease progression markers), patient and informal caregivers’ quality of life, care perceptions and/or satisfaction, health‐care use and costs.

Finally, the target population domain (domain 4 in Table 4) is defined by three subdomains, namely, the type of recipient (patient or informal/family caregiver), condition‐related characteristics (time since diagnosis, disease severity, comorbidity/multimorbidity) and socioeconomic/demographic characteristics (socioeconomic status, cultural group, health literacy, age, sex, living situation).

## 4 DISCUSSION

We developed and validated a 132‐component SMI taxonomy consisting of four domains, 25 subdomains and 103 elements, following an iterative discussion process and a two‐round Delphi approach. The SMI taxonomy is designed as a generic model that can be used as a framework for research, practice and policies for the self‐management of long‐term conditions and that can also be tailored to specific diseases.

This new taxonomy differs from existing taxonomies by its inclusion of additional intervention characteristics that are important for the design, implementation, evaluation and reporting of SMIs. Besides self‐management support techniques, it includes new domains for further specification of the target population, self‐management behaviours and outcomes for measuring SMIs.

The SMI taxonomy should provide a common framework and language to serve as a starting point for the design of primary and secondary studies, as a blueprint for reporting and properly comparing the results of SMI studies and as input to policy debates and the implementation of SMIs.

Having a common language should also allow for more informed decision making and conclusions about necessary SMI components and their effectiveness and, ultimately, contribute to better and more tailored self‐management support for and empowerment of people living with long‐term health conditions.

The fact that three of the four top‐level domains (intervention characteristics, expected patient/caregiver's self‐management behaviours and outcomes for measuring SMIs) scored highest in round 2 of the Delphi survey shows just how important experts feel it is to contemplate a wide array of factors when designing and evaluating SMIs, that is, not to solely address SMI characteristics. The high scores for other levels of the taxonomy probably reflect frequency in some cases (common components would be expected to be rated highly) and perceived importance in other cases (eg support from nurses and primary care staff, adherence to established goals, physical and emotional health, social functioning, self‐care skills, quality of life and health literacy). Components that were eliminated or were rated as having less importance may have been seen as overlapping with other components or as relatively unfamiliar (eg interventions provided by laypersons or population‐based interventions) or simply may not have been clearly expressed.

### 4.1 Links to the existing literature

This research advances the current state of the art by developing a comprehensive taxonomy of key aspects of SMIs as identified and validated by experts and patient representatives.

Our SMI taxonomy ties in with the existing literature in numerous ways. First and foremost, the Practical Reviews in Self‐Management Support (PRISMS) taxonomy published by Pearce et al (2015)^7^ served as an invaluable reference in guiding content development for our self‐management support technique subdomain. However, we introduced one important modification, namely, that we distinguish support techniques from expected self‐management behaviours, as our experience with two European‐funded projects^2^, ^58^ has taught us that the effectiveness of SMIs for long‐term conditions varies according to the disease and to expected behaviours. This differentiation provided important insights into both processes involved in improving the self‐management and more generic and disease‐specific components of our taxonomy. While the PRISMS study also recognized the need to consider other ‘over‐arching dimensions’ such as mode of delivery, personnel, targeting and intensity, it did not provide a structure for these dimensions, as it focuses exclusively on SMI support—a gap our taxonomy fills.

Michie et al^9^, ^11^ devised two theory‐linked taxonomies of behaviour change techniques, the most recent one (CALOR‐RE)^9^ focusing specifically on the structure of interventions designed to help people change physical activity and eating behaviours. Both taxonomies were particularly useful in helping us decide on the content of our support technique subdomain, particularly concerning elements associated with behavioural change in lifestyle‐related behaviours. Although both taxonomies focus on techniques for behavioural change, the authors acknowledge the importance of addressing associated components, including mode of delivery. While the original taxonomies did not include additional components, the research team is currently working on a taxonomy that will include additional dimensions applicable to techniques aimed at behavioural change.^59^

Although the disease management taxonomy of Krumholz et al^10^ has a broader focus than self‐management, it contributed substantially to the general structural design of our taxonomy. Key insights into the literature on self‐management were provided by the review of self‐management approaches for people with long‐term conditions by Barlow et al,^5^ who also provided an interesting synthesis of the wide range of existing interventions and domains. Finally, our research was greatly enhanced by the results of the PRO‐STEP^2^ project, which involved an analysis of 257 systematic reviews on the effectiveness of SMIs, as the PRO‐STEP SMI classification was used as a starting point for our taxonomy.

### 4.2 Strengths and limitations

The SMI taxonomy presented here has several strengths. First, it incorporates key dimensions missing from existing taxonomies that focus mostly on self‐management support techniques. The new components include, among others, mode of delivery, location, provider type, target population, self‐care behaviours and outcomes for measuring SMIs. We believe that this more comprehensive structure, justified by the holistic nature of SMIs, should prompt researchers and other stakeholders to consider multiple aspects of SMIs during intervention design, implementation and evaluation and also to document them in such a way as to make proper comparison across interventions feasible. Second, the initial list of components presented to the expert Delphi panel was based on existing taxonomies and classifications and on the results of the PRO‐STEP project (based on 257 systematic reviews of the effectiveness of SMIs). The preliminary version of the taxonomy, based as it was on a detailed tried‐and‐tested SMI classification system, thus provided a robust starting point. Third, the taxonomy was reviewed and validated in two Delphi rounds by 27 self‐management and taxonomy experts from various relevant backgrounds. Finally, the structuring of the taxonomy into different levels can also be considered a strength, as it will enable stakeholders to design and implement SMIs from a macro‐ to a microperspective.

One of the limitations of our study is that only 16% of the experts that were contacted to participate in the Delphi process responded to the invitation. One of the most plausible explanations for this response rate is that we used e‐mail addresses listed for corresponding authors and some of the addresses from older studies may no longer have been active. Of those that responded, 85% completed round 1 and 81% round 2. The relatively short duration of the field test (two months) limited our ability to explore databases to locate updated addresses or detect periods of holidays or leave. Nevertheless, the Delphi technique is considered to be a suitable consensus‐building method even with a limited number of experts.^60^ In addition, findings from small expert panels in well‐defined knowledge areas have been found to be stable compared with findings by larger samples of experts from the same area of expertise.^61^ A final mitigating factor is that the 27 experts who participated in the panel have a mean of 9.64 years’ experience in the field of self‐management or taxonomy, while some of them are very well recognized at the international level.

### 4.3 Implications for practice and further research

Self‐management is a growing field of interest, for patients, providers and policymakers, as a key component of patient‐centred care.^62^ A vast number of research and implementation projects are currently developing different SMIs. We believe that our SMI taxonomy will make a significant contribution to research in the field and will help improve clinical practice by providing a clear structure for SMI categorization. The SMI taxonomy can be used by researchers, clinicians, policymakers and other stakeholders in various ways: to categorize and develop SMIs using standardized concepts, to translate evidence on SMIs for long‐term conditions into practice, to design and classify SMIs and relevant data in health‐care organization systems, to analyse existing literature, to facilitate comparative effectiveness among SMIs and to characterize a broad range of SMIs. Additionally, on the basis of the SMI taxonomy, reporting standards can be defined that further ensure homogeneous and accurate reporting of SMIs.

The SMI taxonomy is a resource for intervention designers, researchers, practitioners, systematic reviewers and all those wishing to communicate effectively about the content of SMIs. We consider that this taxonomy might also be useful as a reporting framework for future systematic reviews. We realize, however, that our SMI taxonomy, as developed, is quite extensive and complex, suggesting that some guidance is needed to use it for different purposes. We therefore plan to develop a training tool to aid use of the taxonomy in practice.

Note that we deliberately chose not to include in the taxonomy contextual factors and SMI intensity (the duration of intervention delivery and other aspects such as the number, frequency and duration of sessions or types of treatment/dosing schedules). Although these aspects are clearly necessary to obtain a full picture, they reflect the intervention strategy as implemented, and as such, we consider such aspects to be applicable to all types of complex interventions, not just SMIs.^63^, ^64^

Finally, it is important to highlight that the outcomes for measuring SMI domain of the SMI taxonomy feature general examples for long‐term conditions, broadly speaking; for detailed analysis or design purposes, it would be necessary to complement these with core outcomes applicable to specific conditions (Box 1).

BOX 1 **Key messages**

What is already known?

- Self‐management interventions (SMIs) are supportive interventions aimed at increasing patients’ skills and confidence in their ability to manage long‐term conditions.
- SMIs are complex and mostly multicomponent interventions that reflect key elements of patient‐centred care for long‐term conditions.
- The great variability in SMI design and how their components are reported or measured hinders their comparability in terms of effectiveness and cost‐effectiveness.
- While previous studies have proposed SMI taxonomies, they focus mainly on the type of support and pay little attention to other relevant components. No existing taxonomy has been developed on the basis of validation by experts or patient representatives.

What does this study add?

- We have developed and validated a comprehensive consensus‐based taxonomy of SMIs for patients with long‐term conditions.
- The SMI taxonomy provides a framework to characterize in detail four main domains: intervention characteristics, expected patient/caregiver self‐management behaviours, outcomes for measuring SMIs and target population.
- The SMI taxonomy potentially represents a useful guide to stakeholders in the design, implementation, comparison and evaluation of SMIs.
- The SMI taxonomy can enhance the quality of reporting in primary and secondary research in this field.

How might this affect practice?

- The expectation is a better translation of evidence on SMIs to practice, with a consequent improvement in related outcomes for patients.

## CONFLICT OF INTEREST

The authors declare that there is no conflict of interest.

## AUTHOR CONTRIBUTIONS

Carola Orrego, Marta Ballester Monique Heymans, Oliver Groene and Rosa Sunol have made substantial contributions to conception and design. Carola Orrego, Marta Ballester, Estela Camus and Rosa Sunol have made substantial contribution on acquisition of the data. All the authors participated have made substantial contribution to the interpretation of data; have been involved in drafting the manuscript or revising it critically for important intellectual content; and gave final approval of the version to be published. All the authors have participated sufficiently in the work to take public responsibility for appropriate portions of the content and agreed to be accountable for all aspects of the work in ensuring that questions related to the accuracy or integrity of any part of the work are appropriately investigated and resolved.

## Supporting information

Table S1‐3

## Data Availability

All relevant data are within the manuscript and its Supplementary Information files.
