# Supplementary material for: Talking the same language on patient empowerment: Development and content validation of a taxonomy of self‐management interventions for chronic conditions
Source: Health Expect. 2021 Jul 12;24(5):1626–38. doi: 10.1111/hex.13303 (PMC8483213; doi:10.1111/hex.13303)
Supplement: Supplementary file 1 — Table S1‐3 [file HEX-24-1626-s001.docx]

**Supporting information**

**S1- Final Taxonomy of Self-management Intervention (all domains)**

|  |  |  |
| --- | --- | --- |
| **Item level** | **Code** |  |
| **Dom** | **1** | **Self-management intervention characteristics** |
| Subdomain | 1.1 | Self-management Support techniques |
| Element |  | Sharing information |
| Element |  | Skills training |
| Element |  | Stress and/or emotional management |
| Element |  | Shared decision-making |
| Element |  | Goal setting and action planning |
| Element |  | Problem solving skills enhancement |
| Element |  | Self-monitoring training and feedback |
| Element |  | Using prompts and reminders |
| Element |  | Encouraging the use of services |
| Element |  | Providing equipment |
| Element |  | Social support |
| Element |  | Coaching and motivational interviewing |
| Subdomain | 1.2 | Delivery methods |
| Subdomain | 1.2.1 | Type of encounter |
| Element |  | Clinical visit |
| Element |  | Support session |
| Element |  | Self-guided intervention |
| Subdomain | 1.2.2 | Support delivery mode |
| Subdomain | 1.2.2.1 | Face-to-face intervention |
| Subdomain | 1.2.2.2 | Distance/remote intervention |
| Element |  | Phone (calls only) |
| Element |  | Smartphone (text messages, apps…) |
| Element |  | Internet (e-mail, web-based…) |
| Element |  | Specific devices |
| Subdomain | 1.2.3 | Time of communication |
| Element |  | Synchronous |
| Element |  | Asynchronous |
| Subdomain | 1.3 | Recipient |
| Element |  | Individual |
| Element |  | Group |
| Element |  | Specific population |
| Subdomain | 1.4 | Provider Type |
| Element |  | Physician |
| Element |  | Nurse |
| Element |  | Pharmacist |
| Element |  | Physiotherapist |
| Element |  | Occupational therapist |
| Element |  | Social worker |
| Element |  | Psychologist |
| Element |  | Dietician/nutritionist |
| Element |  | Healthcare assistant |
| Element |  | Peer |
| Element |  | Layperson |
| Element |  | Service |
| Subdomain | 1.5 | Location |
| Element |  | Hospital (inpatient care) |
| Element |  | Long-term care centre/nursing home |
| Element |  | Community-based care |
| Element |  | Home care |
| Element |  | Primary care |
| Element |  | Outpatient setting |
| Element |  | Workplace |
| **Dom** | **2** | **Expected patient (or caregiver) self-management behaviours** |
| Subdomain | 2.1 | Lifestyle-related |
| Element |  | Eating behaviours |
| Element |  | Physical activity/exercise |
| Element |  | Smoking cessation or reduction |
| Element |  | Cessation or reduction of the consumption of alcohol or other harmful substances |
| Element |  | Healthy sleep habits |
| Subdomain | 2.2 | Clinical management |
| Element |  | Condition-specific behaviours |
| Element |  | Self-monitoring |
| Element |  | Medication use and adherence |
| Element |  | Early recognition of symptoms |
| Element |  | Asking for professional help or emergency care when needed |
| Element |  | Device management |
| Element |  | Physical management |
| Subdomain | 2.3 | Psychological management |
| Element |  | Handling/managing emotions |
| Subdomain | 2.4 | Social management |
| Element |  | Fitting in at work |
| Element |  | Social roles |
| Element |  | Being able to work |
| Subdomain | 2.5 | Working with healthcare and/or social care providers |
| Element |  | Communicating with healthcare and/or social care providers |
| **Domain** | **3** | **Outcomes for measuring SMIs** |
| Subdomain | 3.1 | Basic empowerment |
| Element |  | Level of knowledge |
| Element |  | Level of health literacy |
| Element |  | Level of skill acquisition |
| Element |  | Level of self-efficacy |
| Element |  | Level of patient activation |
| Subdomain | 3.2 | Adherence to self-management behaviours |
| Element |  | Lifestyle-related behaviours |
| Element |  | Clinical self-management behaviours |
| Element |  | Psychological self-management behaviours |
| Element |  | Social self-management behaviours |
| Element |  | Interactions and communication with healthcare and/or social care providers |
| Subdomain | 3.3 | Clinical outcomes |
| Element |  | Disease progression (clinical markers, symptoms) |
| Element |  | Complications |
| Element |  | Adverse events |
| Element |  | Mortality |
| Subdomain | 3.4 | Patient/caregivers’ quality of life |
| Element |  | Overall quality of life |
| Element |  | Physical functioning |
| Element |  | Psychological and emotional role functioning |
| Element |  | Social functioning |
| Element |  | Sexual functioning |
| Element |  | Burden of treatment |
| Subdomain | 3.5 | Care perception / satisfaction |
| Element |  | Overall satisfaction with self-management intervention |
| Element |  | Perception of being well and sufficiently informed (quality of information provision) |
| Element |  | Perception of patient-provider relationship |
| Element |  | Personalised care |
| Subdomain | 3.6 | Healthcare use |
| Element |  | Type and number of visits |
| Element |  | Hospital admissions and readmissions |
| Element |  | Emergency care |
| Subdomain | 3.7 | Costs |
| Element |  | Healthcare costs for patient |
| Element |  | Healthcare costs |
| Element |  | Direct non-medical costs |
| Element |  | Societal costs |
| **Dom** | **4** | **Target population** |
| Subdomain | 4.1 | As defined by intervention recipient |
| Element |  | Patients |
| Element |  | Informal caregivers or family caregivers |
| Subdomain | 4.2 | As defined by disease-related characteristics |
| Element |  | Time since diagnosis |
| Element |  | Disease severity |
| Element |  | Comorbidity and multimorbidity |
| Subdomain | 4.3 | As defined by socioeconomic / demographic characteristics |
| Element |  | Socioeconomic status |
| Element |  | Cultural group |
| Element |  | Health literacy |
| Element |  | Digital literacy |
| Element |  | Biological sex or gender |
| Element |  | Age |
| Element |  | Living situation |

**S2. Overall history of changes through Delphi rounds and final decisions**

| **Delphi results for round I and II and final decision** | | | | | | | | | | | |  |
| --- | --- | --- | --- | --- | --- | --- | --- | --- | --- | --- | --- | --- |
|  | | **Round I** | |  | **Round II** | | | **Changes** | | **Final decision** | |  |
| **Item level** | **Taxonomy** | **Mean** | **SD** |  | **Mean** | **SD** | **Median** | |  | |  | |
| Domain | Intervention characteristics | 8.88 | 0.33 |  | 8.93 | 0.26 | 9 | |  | | Include | |
| Subdomain | Self-management support techniques | 8.48 | 1.42 |  | 8.67 | 0.47 | 9 | |  | | Include | |
| Element | Sharing information | 8.31 | 1.21 |  | 8.45 | 1.04 | 9 | |  | | Include | |
| Element | Skills training | 8.47 | 0.88 |  | 8.59 | 0.77 | 9 | |  | | Include | |
| Element | Stress and/or emotional management | 8 | 1.19 |  | 8.28 | 0.64 | 8 | |  | | Include | |
| Element | Shared decision-making | 8.09 | 1.36 |  | 8.31 | 0.83 | 9 | |  | | Include | |
| Element | Goal setting and action planning | 8.35 | 1.08 |  | 8.31 | 1.12 | 9 | |  | | Include | |
| Element | Enhancing problem solving skills | 8.29 | 1.02 |  | 8.38 | 0.89 | 9 | |  | | Include | |
| Element | Self-monitoring training and feedback | 8.26 | 1.17 |  | 8.21 | 0.9 | 8 | | Modified^1^ | | Include | |
| Element | Using prompts and reminders | 7.47 | 1.72 |  | 7.72 | 1.31 | 8 | |  | | Include | |
| Element | Encouraging use of services | 7.09 | 2.08 |  | 7.59 | 1.38 | 8 | | Modified after round II ^1^ | | Include | |
| Element | Provision of equipment | 7.25 | 1.89 |  | 7.24 | 1.43 | 7 | |  | | Include | |
| Element | Social support | 7.94 | 1.35 |  | 8.17 | 0.91 | 8 | |  | | Include | |
| Element | Coaching and motivational interviewing |  |  |  | 7.75 | 0.99 | 8 | | Merged ^2^ | | Include | |
| Subdomain | Support delivery methods | 8.55 | 0.86 |  | 8.67 | 0.61 | 9 | | Modified^1^ | | Include | |
| Subdomain II | Type of encounter | 7.63 | 2.09 |  | 8.12 | 1.01 | 8 | |  | | Include | |
| Element | Clinical visits | 7.76 | 1.67 |  | 8.14 | 0.82 | 8 | | Modified^1^ | | Include | |
| Element | *Educational and/or training sessions* | 7.85 | 1.67 |  | -- | -- | -- | | Eliminated | | Eliminate | |
| Element | Support sessions | 7.85 | 1.65 |  | 8.24 | 0.57 | 8 | |  | | Include | |
| Element | Self-guided | 6.9 | 1.92 |  | 7.29 | 1.22 | 7 | | Modified^1^ | | Include | |
| SubdomainII | Mode | 7.88 | 1.75 |  | 8.04 | 1.14 | 8 | | Modified^1^ | | Include | |
| SubdomainIII | Face-to-face interventions | 8.38 | 0.78 |  | 8.38 | 0.68 | 8.5 | |  | | Include | |
| SubdomainIII | Distance or Remote interventions | 8 | 1.12 |  | 8.12 | 0.93 | 8 | | Modified^1^ | | Include | |
| Element | Phone (only for calls) | 7.76 | 1.42 |  | 7.83 | 1.12 | 8 | |  | | Include | |
| Element | Smart phone (SMS, apps…) | 7.88 | 1.17 |  | 8.03 | 0.93 | 8 | |  | | Include | |
| Element | Internet - based (e-mail, web…) | 7.79 | 1.41 |  | 7.79 | 1 | 8 | |  | | Include | |
| *Element* | *Mail* | 6.15 | 2.27 |  | -- | -- | -- | | Eliminated | | Eliminate | |
| Element | Using a specific device | 7.21 | 1.92 |  | 7.03 | 1.81 | 7 | |  | | Include | |
| *SubdomainIII* | *Physical educational materials* | 7.56 | 1.73 |  | -- | -- | -- | | Eliminated | | Eliminate | |
| SubdomainII | Time of communication | 7.84 | 1.25 |  | 7.81 | 1.09 | 8 | | Modified^1^ | | Include | |
| Element | Synchronous | 8.27 | 0.99 |  | 8.28 | 0.87 | 8 | |  | | Include | |
| Element | Asynchronous | 7.85 | 1.56 |  | 7.97 | 1.19 | 8 | |  | | Include | |
| SubdomainII | Recipient | 7.88 | 1.48 |  | 7.69 | 1.7 | 8 | | Modified^1^ | | Include | |
| Element | Specific Population |  |  |  | 7.21 | 1.61 | 7.5 | | Added | | Include | |
| Element | Groups | 8.12 | 1.35 |  | 8.14 | 1.2 | 8 | |  | | Include | |
| Element | Individual | 8.32 | 0.9 |  | 8.55 | 0.5 | 9 | |  | | Include | |
| Subdomain | Type of provider | 8.15 | 1.48 |  | 8.19 | 0.86 | 8 | |  | | Include | |
| Element | Physicians | 7.97 | 1.68 |  | 8.1 | 1.16 | 8 | |  | | Include | |
| Element | Nurse | 8.64 | 0.54 |  | 8.45 | 1 | 9 | |  | | Include | |
| Element | Pharmacist | 7.97 | 1.31 |  | 8 | 1.17 | 8 | |  | | Include | |
| Element | Physiotherapist | 8.19 | 0.98 |  | 8.04 | 1.12 | 8 | |  | | Include | |
| Element | Occupational therapist | 8.16 | 1 |  | 8 | 1.07 | 8 | |  | | Include | |
| Element | Social worker | 7.91 | 1.44 |  | 8 | 1.05 | 8 | |  | | Include | |
| Element | Psychologist | 8.18 | 1.09 |  | 8.14 | 1.07 | 8 | | Modified after round II^4^ | | Include | |
| Element | Dietician/nutritionist | 8.15 | 1.16 |  | 8.14 | 1.12 | 8.5 | |  | | Include | |
| Element | Healthcare assistant |  |  |  |  |  |  | | Added after round II | |  | |
| Element | Peers | 8.16 | 1.55 |  | 8.07 | 1.41 | 8 | |  | | Include | |
| Element | Lay person | 7.52 | 1.85 |  | 7.14 | 2 | 8 | |  | | Include | |
| Element | Service | 6.97 | 2.1 |  | 7.14 | 1.55 | 7 | |  | | Include | |
| Subdomain | Location | 8.27 | 1.42 |  | 8.19 | 1.12 | 8 | |  | | Include | |
| Element | Hospital care | 7.52 | 1.67 |  | 7.79 | 1 | 8 | |  | | Include | |
| Element | Long-term care centres and nursing homes | 7.34 | 1.78 |  | 7.64 | 0.97 | 8 | |  | | Include | |
| Element | Community-based care | 8.47 | 1.01 |  | 8.76 | 0.5 | 9 | |  | | Include | |
| Element | Homecare | 8.41 | 0.94 |  | 8.55 | 0.77 | 9 | |  | | Include | |
| Element | Primary care | 8.41 | 0.91 |  | 8.62 | 0.61 | 9 | |  | | Include | |
| Element | Outpatients care | 8.03 | 1.32 |  | 8.21 | 0.85 | 8 | |  | | Include | |
| Element | Workplace | 7.45 | 1.46 |  | 7.62 | 1.24 | 8 | |  | | Include | |
| *Element* | *Emergency care* | 6.12 | 2.77 |  | -- | -- | -- | | Eliminated | | Eliminate | |
| Domain | Expected patient (or carer) self-management behaviours | 8.76 | 0.55 |  | 8.85 | 0.45 | 9 | |  | | Include | |
| Subdomain | Lifestyle related behaviours | 8.42 | 0.92 |  | 8.52 | 0.69 | 9 | |  | | Include | |
| Element | Eating behaviours | 8.38 | 1.16 |  | 8.37 | 0.95 | 9 | |  | | Include | |
| Element | Physical activity/exercise | 8.5 | 1.01 |  | 8.68 | 0.66 | 9 | |  | | Include | |
| Element | Smoking cessation or reduction | 8.24 | 1.35 |  | 8.43 | 0.94 | 9 | |  | | Include | |
| Element | Alcohol consumption, and other harmful consumptions, cessation or reduction | 8.12 | 1.35 |  | 8.25 | 1.06 | 9 | | Modified^1^ | | Include | |
| Element | Healthy sleep behaviours |  |  |  | 7.65 | 1.66 | 8 | | Added | | Include | |
| Subdomain | Clinical management | 8.31 | 1.13 |  | 8.33 | 0.86 | 9 | |  | | Include | |
| Element | Condition-specific behaviours | 8.52 | 0.96 |  | 8.54 | 0.73 | 9 | |  | | Include | |
| Element | Self-monitoring | 8.56 | 1.14 |  | 8.52 | 0.92 | 9 | |  | | Include | |
| Element | Medication use and adherence | 8.21 | 1.35 |  | 8.25 | 1.18 | 9 | |  | | Include | |
| Element | Early recognition of symptoms | 8.21 | 1.41 |  | 8.26 | 1.04 | 9 | |  | | Include | |
| Element | Asking for professional help or emergency care when needed | 8.18 | 1.52 |  | 8.46 | 0.78 | 9 | | Modified^1^ | | Include | |
| Element | Managing devices | 8.09 | 1.31 |  | 8.15 | 1.11 | 8 | |  | | Include | |
| Element | Physical management | 8.26 | 1.48 |  | 8.46 | 0.63 | 9 | |  | | Include | |
| Subdomain | Psychological management | 8.39 | 0.81 |  | 8.3 | 0.97 | 9 | |  | | Include | |
| Element | Handling /managing emotions | 8.15 | 1.58 |  | 8.5 | 0.64 | 9 | |  | | Include | |
| Subdomain | Social management | 8.27 | 0.9 |  | 8.22 | 0.99 | 8 | |  | | Include | |
| Element | Fitting in at work | 7.93 | 1.3 |  | 7.64 | 1.2 | 8 | |  | | Include | |
| Element | Social roles | 8.1 | 1.25 |  | 7.93 | 1.07 | 8 | |  | | Include | |
| Element | Being able to work | 7.8 | 1.3 |  | 7.57 | 1.66 | 8 | |  | | Include | |
| Subdomain | Working with healthcare and/or social care providers | 7.79 | 1.34 |  | 8.04 | 1 | 8 | | Modified after round I & II^1^ | | Include | |
| Element | Communication with healthcare and/or social care providers | 8.06 | 1.58 |  | 8.36 | 0.89 | 9 | | Modified after round II | | Include | |
| Domain | Type of outcomes to measure self-management interventions | 8.82 | 0.39 |  | 8.93 | 0.26 | 9 | | Modified after round II | | Include | |
| Subdomain | Empowerment basic components | 8.24 | 1.61 |  | 8.37 | 0.73 | 8 | |  | | Include | |
| Element | Level of knowledge | 8.38 | 1.26 |  | 8.54 | 0.68 | 9 | |  | | Include | |
| Element | Level of health literacy | 7.82 | 1.85 |  | 8.43 | 0.82 | 9 | |  | | Include | |
| Element | Level of skill acquisition | 8.35 | 1.21 |  | 8.46 | 0.68 | 9 | | Modified^1^ | | Include | |
| Element | Level of self-efficacy | 8.38 | 1.19 |  | 8.43 | 1.08 | 9 | |  | | Include | |
| Element | Patient activation level | 7.73 | 2.26 |  | 7.57 | 2.03 | 8.5 | |  | | Include | |
| Subdomain | Level of adherence of the expected self-management behaviours | 7.94 | 1.64 |  | 8.07 | 0.86 | 8 | |  | | Include | |
| Element | Lifestyle related behaviours | 7.91 | 1.84 |  | 8.25 | 0.78 | 8 | |  | | Include | |
| Element | Clinical self-management behaviours | 7.85 | 1.92 |  | 8.29 | 0.84 | 8 | | Modified^1^ | | Include | |
| Element | Psychological management level | 8.34 | 1.51 |  | 8.5 | 0.63 | 9 | |  | | Include | |
| Element | Social management level | 7.97 | 1.72 |  | 8.21 | 0.94 | 8 | |  | | Include | |
| Element | Interactions and communication with healthcare and/or social care providers | 7.91 | 1.58 |  | 8.21 | 0.67 | 8 | | Modified^1^ | | Include | |
| Subdomain | Clinical outcomes | 8.44 | 0.98 |  | 8.56 | 0.68 | 9 | | Modified^1^ | | Include | |
| Element | Progression of disease (clinical markers, symptoms) | 7.97 | 1.2 |  | 8.04 | 0.98 | 8 | |  | | Include | |
| Element | Complications | 8.09 | 1.09 |  | 8.08 | 0.92 | 8 | | Modified after round I & II^1^ | | Include | |
| Element | Adverse events | 8.03 | 1.19 |  | 7.86 | 1.64 | 8 | |  | | Include | |
| Element | Mortality | 7.82 | 1.4 |  | 7.75 | 1.72 | 8 | |  | | Include | |
| Subdomain | Quality of life of patients and caregivers | 8.53 | 0.95 |  | 8.59 | 0.83 | 9 | |  | | Include | |
| Element | Overall quality of life | 8.47 | 1.06 |  | 8.46 | 1.05 | 9 | |  | | Include | |
| Element | Physical functioning | 8.5 | 0.88 |  | 8.46 | 0.91 | 9 | |  | | Include | |
| Element | Psychological and emotional role functioning | 8.53 | 0.75 |  | 8.64 | 0.55 | 9 | | Modified^1^ | | Include | |
| Element | Social functioning | 8.42 | 0.82 |  | 8.33 | 0.82 | 8 | | Split after round II^3^ | | Include | |
| Element | Sexual functioning |  |  |  |  |  |  | | Split after round ii^3^ | |  | |
| Element | Burden of treatment | 8.44 | 0.85 |  | 8.59 | 0.49 | 9 | |  | | Include | |
| Subdomain | Perceptions and/or satisfaction with care | 8.12 | 1.34 |  | 7.85 | 1.76 | 8 | |  | | Include | |
| Element | Overall satisfaction with self-management interventions | 7.76 | 1.63 |  | 7.63 | 1.16 | 8 | |  | | Include | |
| Element | Perception of being well and enough informed (quality of information provision) | 7.97 | 1.47 |  | 7.93 | 1.68 | 8 | | Modified^1^ | | Include | |
| Element | Perception on patient - provider relationship | 8.38 | 0.77 |  | 8.3 | 0.66 | 8 | |  | | Include | |
| Element | Personalized care | 8.09 | 1.18 |  | 8.11 | 1.52 | 8 | |  | | Include | |
| Subdomain | Healthcare use | 8.06 | 1.35 |  | 8.15 | 1.01 | 8 | |  | | Include | |
| Element | Type and number of visits | 7.81 | 1.72 |  | 7.69 | 1.41 | 8 | |  | | Include | |
| Element | Hospital admissions and readmissions | 7.88 | 1.53 |  | 7.88 | 0.93 | 8 | |  | | Include | |
| Element | Emergency care | 7.84 | 1.57 |  | 7.72 | 1.66 | 8 | |  | | Include | |
| Subdomain | Cost | 8 | 1.21 |  | 7.93 | 1.05 | 8 | |  | | Include | |
| Element | Healthcare costs for patient | 7.97 | 1.12 |  | 8.04 | 0.85 | 8 | |  | | Include | |
| Element | Healthcare costs | 7.88 | 1.62 |  | 7.92 | 1.41 | 8 | |  | | Include | |
| Element | Direct non-medical costs |  |  |  | 7.92 | 1.24 | 8 | | Added | | Include | |
| Element | Societal costs | 7.82 | 1.59 |  | 8.04 | 1.17 | 8 | |  | | Include | |
| Domain | Target population | 8.33 | 1.63 |  | 8.41 | 1.25 | 9 | |  | | Include | |
| Subdomain | Defined by who receives the intervention | 8.39 | 1.01 |  | 8.23 | 0.97 | 9 | | Modified after round II^1^ | | Include | |
| Element | Patients | 8.68 | 0.87 |  | 8.81 | 0.62 | 9 | |  | | Include | |
| Element | Informal caregivers or family caregivers | 8.24 | 1.14 |  | 8.31 | 0.91 | 9 | |  | | Include | |
| *Element* | *Community* | 7.94 | 1.46 |  | -- | -- | -- | |  | | Eliminate | |
| Subdomain | Defined by disease related characteristics | 8.36 | 0.95 |  | 8.35 | 0.78 | 9 | |  | | Include | |
| Element | Time since diagnosis | 7.81 | 1.83 |  | 7.88 | 1.6 | 8 | |  | | Include | |
| Element | Disease severity | 7.97 | 1.47 |  | 7.88 | 1.31 | 8 | |  | | Include | |
| Element | Comorbidity and multi-morbidity | 8.16 | 1.18 |  | 8.38 | 0.74 | 8.5 | | Modified^1^ | | Include | |
| Subdomain | Defined by socio-economic or demographic characteristics | 8.03 | 1.42 |  | 8.12 | 1.19 | 8.5 | |  | | Include | |
| Element | Socioeconomic status | 7.85 | 1.6 |  | 7.93 | 1.39 | 8 | |  | | Include | |
| Element | Cultural groups | 7.65 | 1.94 |  | 7.81 | 1.47 | 8 | | Modified after round II | | Include | |
| Element | Health literacy | 8 | 1.37 |  | 8.15 | 1.18 | 9 | |  | | Include | |
| Element | Digital literacy |  |  |  | 7.54 | 1.15 | 8 | | Added | | Include | |
| Element | Biological sex or gender | 7.5 | 1.93 |  | 7.48 | 1.29 | 8 | | Modified^1^ | | Include | |
| Element | Age | 8 | 1.39 |  | 7.93 | 1.25 | 8 | |  | | Include | |
| Element | Living situation | 7.62 | 1.55 |  | 7.78 | 1.13 | 8 | |  | | Include | |

**S3.** Taxonomy of Self-Management Interventions: final definitions and examples.

| **Item level** | **Taxonomy** | **Definition/explanation/examples** | **References** |
| --- | --- | --- | --- |
| Domain | Intervention characteristics | The first of the four domains (top levels) of the taxonomy. It is composed of different components that may act independently or interdependently to improve self-management behaviours. These components are **self-management support techniques, support delivery methods, provider type, and location**. They can affect **outcomes** at a patient, organizational/service, or population level (or all of these in some cases) for a specific **type of expected behaviour** and **target population.** |  |
| Subdomain | Self-management support techniques | Techniques or methods used to provide care and encouragement to patients with chronic illness and their caregivers to help them understand that they can play a central role in managing their illness, make informed decisions about their care, and engage in appropriate behaviours. | ^1^ |
| Element | Sharing information | Providing and sharing information and explanations about self-management topics, such as possible or likely general consequences of disease management, costs and benefits, and information about what other people are doing, about the disease itself, or about any other relevant aspects that could lead to improved self-management. This information may be accompanied by printed material.  Examples: Educational session on healthy eating for people with obesity, provision of a printed leaflet on the importance of foot care in diabetes, or a link to a website with information on chronic obstructive pulmonary disease care. | ^2,3^ |
| Element | Skills training | Teaching practical skills and techniques to support everyday activities, with hands-on practice and continuous assessment of skills and techniques learned.  Examples: Tips on inhaler use, foot care. | ^4,5^ |
| Element | Stress and/or emotional management | Helping patients understand the role of stress and emotions and teaching them to use different coping strategies to manage, for example, stress and painful emotions caused by their disease.  Examples: Mindfulness, exercise, stretching, listening to music, deep breathing, meditation. |  |
| Element | Shared decision-making | Involving patients as active partners with a provider or multidisciplinary team to encourage and facilitate shared decision-making processes. Within these processes, partners explore different care or treatment options, discuss the risks and benefits of each, and reach a joint decision.  Shared decision-making is appropriate for any situation where there is more than one reasonable course of action and where no single option is self-evidently best for everyone.  Examples: Discussion of different treatments, procedures, diagnostic tests, or healthy lifestyle behaviours and joint decision on which options are best suited to the patient’s needs and preferences. | ^6,7^ |
| Element | Goal setting and action planning | Encouraging patients to set one or more achievable goals based on their needs and preferences. These goals may be behaviours or outcomes and their establishment can be used as a starting point for productive interaction. The process usually involves the formulation of a detailed action plan specifying what the person will do and at least when and/or where they will do it. It could also include joint assessment of behaviours and discussion of goal modifications and the writing up of agreed-on action plans, including plans for emergency situations.  Examples of goals: daily walking distance (e.g., 2 km), weight loss (e.g., X%), diet and exercise. | ^2,4^ |
| Element | Problem-solving skills enhancement | Analysing or prompting a patient to analyse factors that influence their behaviour and generating or selecting strategies to reduce or overcome barriers and/or enhance facilitators. Strategies include anticipation, self-treatment, resource utilization, and problem management. Ideally, there should be an initial plan, but this is not a requisite.  Example: Identification and attenuation of environmental barriers to everyday physical activities. | ^2,8,9^ |
| Element | Self-monitoring training and feedback | Training and encouraging people to recognize, monitor, and record behaviours, symptoms, or clinical data. This process may include regular feedback from a clinician, or a synopsis of information registered in a digital tool to encourage the patient to continue monitoring their illness and behaviours.  Example: Showing a patient how to record blood sugar levels, physical activity, pain. |  |
| Element | Using prompts and reminders | Using environmental and practical prompts to remind patients and caregivers to perform a required behaviour compatible with their preferences. These prompts can also be used to remind patients to avoid an undesirable behaviour or to replace it with a more desirable one. Regular reminders about correct techniques and skills are also important for maintaining high standards.  Examples: Encouraging/teaching a patient to use electronic reminders (e.g., a medication reminder app) or to make a to-do list for daily self-management behaviours. | ^4^ |
| Element | Encouraging the use of services | Stimulating, facilitating, or promoting the use of services that could enhance self-management behaviours. These encompass a wide and varied range of services outside the direct healthcare system and include electronic and community support services.  Examples: Encouraging a patient to use a new service (e.g., attend swimming pool sessions) or to use a given service either more (e.g., a 24-hour telephone support service) or less (e.g., emergency department). | ^4^ |
| Element | Providing equipment | Providing equipment to enable, assist with, or promote self-monitoring and/or self-management of a chronic condition. It requires training in correct usage and adequate assessment of equipment needs and suitability.  Examples: Peak flow meters, pill organizers, pedometers, telemedicine equipment. | ^5^ |
| Element | Social support | Helping a person to think through how they might elicit social support from others to help them achieve behavioural or outcome goals. It could also include the actual provision of social support or discussions about social support networks suited to the patient’s preferences, needs, disease burden, and additional life burdens.  Part of this support includes linking the individual to relevant community services to enhance socialization and make the most of support mechanisms in the local community.  Examples: Encouraging family members to become involved in helping the patient manage their disease or patients to participate in a local exercise group. | ^5,8^ |
| Element | Coaching and motivational interviewing | Facilitating behavioural change in an interpersonal process by identifying an individual's values and core strengths and transforming goals into action using targeted and tailored strategies and support. One provider (healthcare professional, peer or lay person) should be designated as a coach. Motivational interviewing and counselling are included in this element, as collaborative conversations between practitioners and patients can strengthen patient motivation and commitment, minimize resistance, and resolve ambivalence to change.  Examples: Nurse-led coaching sessions to ease the transition from hospital to home, rehabilitation programs using coaching methods. | ^10–12^ |
| Subdomain | Support delivery methods | Subset of intervention characteristics that describe how an intervention is provided; it includes type of encounter, mode of delivery, time of communication, and recipient. |  |
| Subdomain | Type of encounter | Context in which self-management support is provided. The support could be provided as part of a clinical visit or a specific self-management session or it could be self-guided. | ^13^ ^14^ |
| Element | Clinical visit | Provision of self-management support in face-to-face or virtual interactions between a patient and a healthcare professional or team of professionals during routine clinical practice (e.g., follow-up visits or tests). This encounter is not specific to ambulatory care and could also be provided during hospitalization. |  |
| Element | Support session | An encounter specifically designed for providing self-management support. It can involve different types of support, provided alone or in combination. Methods include seminars, workshops, presentations, discussion of information and materials (e.g., posters or flyers), role-play, demonstration sessions, etc. | ^4^ |
| Element | Self-guided intervention | No specific context is designed for this intervention. Patients or caregivers act on their own initiative. |  |
| Subdomain | Support delivery mode | Mode by which the self-management intervention is delivered. |  |
| Subdomain | Face-to-face intervention | Self-management support delivered in a face-to-face encounter between the providers and patients and/or caregivers. |  |
| Subdomain | Distance or Remote interventions | Self-management support delivered remotely, i.e., when the patients and/or caregivers are not in the same place as the providers. This interaction can take place through different communication means or tools (e.g., telephone, smartphone, Internet). |  |
| Element | Phone (calls only) | Self-management support delivered over a landline or mobile phone in a conversation between patients/caregivers and providers. |  |
| Element | Smartphone and tablets (text message, apps…) | Self-management support delivered via a smartphone, tablet, or pocket-sized computer with access to the Internet and software components (apps). |  |
| Element | Internet - based (e-mail, web…) | Self-management support delivered via a technological communication network. It can be asynchronous (e.g., email, web-based forum) or synchronous (e.g., webinar, Skype meeting, or similar). |  |
| Element | Specific device | Use of specifically designed devices such as telemonitoring and telecare systems can facilitate support interactions between providers and patients/caregivers and enhance self-management.  Example: Telemonitoring device. |  |
| Subdomain | Time of communication | Time at which the intervention is provided (synchronous or asynchronous) |  |
| Element | Synchronous | Simultaneous communication in which an interaction occurs in real time.  Examples: Face-to-face encounters, teleconferences, chats. |  |
| Element | Asynchronous | Communication that does not require the provider and patient to be online at the same time.  Example: Online platform where a patient records data every day and receives monthly feedback. |  |
| Subdomain | Recipient | The person or people targeted by a self-management intervention, i.e., those who are expected to manage their chronic condition. Depending on the intervention, the recipients may be individuals, groups, or a general population.  Examples: Personalized shared decision-making intervention (individual), peer support group (group), national public health campaign (general population). |  |
| Element | Individual | A single person receive the self-management support.  Examples: Self-guided actions or support during a clinical visit or within the context of a support or educational session. |  |
|  |  |  |  |
| Element | Group | Two or more patients or caregivers brought together to receive a self-management intervention. Group interventions are normally organized for efficiency purposes or to facilitate learning and knowledge exchange among peers.  Example: Peer-led education group to enhance physical activity in obese individuals. |  |
|  |  |  |  |
| Element | Specific Population | Broad group of people targeted by a self-management intervention, usually defined by where they live (e.g., city, region, or country). Population-level interventions are typically large-scale and have the potential to improve the health of a population. Other outcomes are also possible.  Example: Public health campaign to raise awareness of type 2 diabetes prevention. | ^15,16^ |
| Subdomain | Provider type | Person or service delivering the intervention. Self-management support can be provided by individuals or multidisciplinary teams. Different practitioners or lay providers could be involved at different stages/levels. |  |
| Element | Physician~~s~~ | Physicians across all levels and specialties, such as surgeons, pulmonologists, cardiologists, family doctors, and psychiatrists. |  |
| Element | Nurse~~s~~ | Nurses across different areas and levels of care (hospitals, primary care centres, home care, etc.). Also, specialist nurses in a wide range of areas (e.g., diabetes education, cardiology, surgical care, rehabilitation). |  |
| Element | Pharmacist | *Self-explanatory* |  |
| Element | Physiotherapist | *Self-explanatory* |  |
| Element | Occupational therapist | Occupational therapists and occupational therapy assistants are healthcare professionals skilled in self-management support, prevention, lifestyle changes, and physical and psychosocial rehabilitation. They help individuals participate in productive and meaningful activities of daily living by working closely with patients and caregivers during the evaluation and intervention process. Occupational therapy is particularly relevant to individuals living with a chronic disease. | ^17^ |
| Element | Social worker | Social workers work with patients in a variety of settings to help them manage, solve, and cope with a range of issues associated with living with a chronic disease. Some patients seek the help of a social worker during times of hardship. | ^18^ |
| Element | Psychologist | *Self-explanatory* |  |
| Element | Dietician/nutritionist | *Self-explanatory* |  |
| Element | Healthcare assistant | Healthcare assistants work in a range of healthcare and community settings under the guidance of qualified healthcare professionals. The nature of their role varies according to where they work, but for self-management purposes their responsibilities could include communication with patients, relatives, and caregiver’s assistance with clinical duties and personal care, and promotion of positive mental/physical/nutritional health. |  |
| Element | Peer | A peer in the context of self-management is someone who shares the experience of living with a (long-term) condition and participates in the provision of the intervention. They usually have the knowledge and skills to manage and solve problems related to the disease in question and share this information to engage and activate other less motivated patients. | ^19^ |
| Element | Lay person | A person without professional or specialized knowledge in healthcare . Lay people usually receive specific training to act as mentors in self-management interventions. They can also collaborate as cultural mediators for specific target groups.  Example: A lay person from a specific ethnic background working as a cultural mediator for people from the same background targeted by an intervention. | ^20^ |
| Element | Service | An institution, organization, association, or service provider that delivers self-management support without interacting between other support providers (professionals/peers/lay people) and recipients (patients, caregivers, general population)  Examples: A national healthcare system that runs a public health campaign or an organization that develops an app for patients to download and use independently. | ^21^ |
| Subdomain | Location | Place, organization, or system in which a self-management intervention is planned, organized, or implemented. Interventions can be implemented with the participation of different levels of care (e.g., home care with support from primary care services in collaboration with specialist outreach from tertiary centres). |  |
| Element | Hospital | Self-management interventions provided during hospitalization. |  |
| Element | Long-term care centres and nursing homes | Self-management interventions provided during a patient’s time at a long-term care centre or nursing home. |  |
| Element | Community-based care | Self-management interventions provided or implemented at a community institution, such as a school, community pharmacy, social centre, community centre, etc. | ^22^ |
| Element | Homecare | Self-management interventions provided or implemented at a patient’s home (e.g., home-care visits, telemedicine, etc.). |  |
| Element | Primary care | Self-management interventions provided or implemented within the primary care system (e.g., primary care centre, GP offices). |  |
| Element | Outpatient care | Self-management interventions provided or implemented by specialists during consultation to an ambulatory visit at hospital or other settings of specialist care. |  |
| Element | Workplace | Self-management interventions provided or implemented at the workplace of the patients |  |
| Domain | Expected patient/caregivers self-management behaviours. | Decisions and behaviours that patients with chronic condition (or their caregivers) engage in that affect their health. They are the focus of self-management support interventions. |  |
| Subdomain | Lifestyle-related behaviours | Lifestyle practices that influence health and determine the impact and severity of a patient’s chronic condition. |  |
| Element | Eating behaviours | Healthy eating habits and the extent to which a person adheres to dietary recommendations agreed on with a healthcare provider. | ^4,23^ |
| Element | Physical activity /exercise | Any activity that gets a patient up and about. It includes everyday activities like walking to the shops, gardening or housework, and leisure activities such as riding a bike, swimming, or playing football. This element also covers the extent to which a person adheres to a physical activity plan agreed on with a healthcare provider. | ^4,24^ |
| Element | Smoking cessation or reduction | Quitting or cutting down on smoking. |  |
| Element | Cessation or reduction of the consumption of alcohol or other harmful substances | Quitting or cutting down on alcohol or other harmful substances. |  |
| Element | Healthy sleep habits | Actions addressed to promote sleep health |  |
| Subdomain | Clinical management | Decisions and actions specifically related to the physical dimension of self-management. Includes self-monitoring of signs and symptoms, medication use, early recognition of symptoms, and physical management of chronic disease. |  |
| Element | Condition-specific behaviours | Activities aimed at achieving better management of a specific condition.  Example: Foot care for patients with diabetes. |  |
| Element | Self-monitoring | Tracking one’s symptoms and or behaviours. In some cases, this may involve subsequent adjustment of behaviours.  Examples: Monitoring of blood sugar levels, peak flow recording, tracking of calorie intake. |  |
| Element | Medication use and adherence | Includes medication use, which refers to the degree to which a patient takes their medication properly (administration, timing, etc.), and medication adherence, which refers to the degree to which a patient adheres to medication use recommendations agreed on with a healthcare provider. For correct medication use and adherence, patients/caregivers need to know how and when a medication should be taken, what should be avoided, and what negative effects should be looked out for. | ^25–30^ |
| Element | Early recognition of symptoms | Understanding of and appropriate and timely monitoring of symptoms. Example: A list of worrisome symptoms, including indications of when it is necessary to contact the doctor’s office or go to the emergency department. |  |
| Element | Asking for professional help or emergency care when needed | The ability of patients or caregivers to ask for professional help or emergency care in an effective and timely manner.,. |  |
| Element | Device Management | Appropriate use of self-management devices, including knowing how to use a device and manage tasks and knowing when and how to ask for technical support or help.  Examples: Use of an insulin pump, telehealth care system. | ^25^ ^31^ |
| Element | Physical management | Coping with pain, tiredness, limited energy, physical impairments, etc. |  |
| Subdomain | Psychological management | Coping with and managing emotions at different stages of illness and applying skills to improve a great variety of situations. | ^32–35^ |
| Element | Handling /managing emotions | Includes reducing and managing stress, boosting positive states, not ruminating, and self-assessment of emotional wellbeing. | ^4^ |
| Subdomain | Social management | Ability to interact with other people and to build relationships in different social situations (e.g., in the community, at work). |  |
| Element | Fitting in at work | Ability to fit in at the workplace and in the work environment |  |
| Element | Social roles | Relationships, volunteering, leisure activities, etc. |  |
| Element | Being able to work | A patient’s ability to enter and stay in the workforce despite their chronic condition. This ability involves, among other things, maintaining a balance between work responsibilities and disease management needs, attending work, executing tasks, meeting deadlines, and maintaining a working relationship with managers and colleagues. |  |
| Subdomain | Working with healthcare and/or social care providers | This sub-domain includes the patients’ capacity and skills to work with health and/or social care providers in an effective and stisfatory manner, |  |
| Element | Communication with healthcare and/or social care providers | This element covers a wide variety of interventions linked to and/or focused on communication as well as interventions designed to improve communication between patients and/or caregivers and healthcare or social care providers. |  |
| Domain | Outcomes to measure the effect of a self-management interventions | This domain includes the subdomains featuring outcome measures to assess the impact of self-management interventions: basic empowerment, fulfilment of expected self-management behaviours, clinical outcomes, patient and carer quality of life, perceptions of and/or satisfaction with care, healthcare use, and cost. |  |
| Subdomain | Basic empowerment components | This subdomain contains basic components of empowerment which include: knowledge, skills, self-efficacy, and patient activation. |  |
| Element | Level of knowledge | Familiarity with or awareness or understanding of facts, information, and descriptions and skills acquired through experience or education involving perceiving, discovering, and learning. |  |
| Element | Level of health literacy | Degree to which individuals are able to obtain, process, and understand basic health information and services in order to make appropriate health decisions. Health literacy can be considered both a moderator of intervention effectiveness and an outcome to be improved. | ^36,37^ |
| Element | Level of skill acquisition | Expertise needed to complete a task. |  |
| Element | Level of self-efficacy | Belief in one’s ability to achieve a goal through specific behaviours. Self-efficacy is one of the key connections between knowledge and action, since belief in one’s ability to execute a behaviour usually precedes one’s attempt at this behaviour. Self-efficacy also affects choice of behaviours, settings in which behaviours are executed, and the effort and persistence behind the performance of a given task. | ^34,35^ |
| Element | Level of patient activation | Degree to which a patient understands their role in the healthcare process and their level of knowledge, skill, and confidence in managing their own health. Patient activation involves four stages: (1) believing the patient has an important role, (2) having the confidence and knowledge necessary to take action, (3) actually taking action to maintain and improve one's health, and (4) staying the course even under stress. | ^38–40^ |
| Subdomain | Level of adherence **to expected** self-management behaviours | This subdomain includes cutcome measures of the fulfilment of expected self-management behaviours related to clinical management, coping with consequences, lifestyle changes, and interactions with professionals and the healthcare system. | ^41,42^ |
| Element | Lifestyle-related behaviours | Outcome measures for assessing patient or carer adherence to expected lifestyle behaviours  Example: number of cigarettes smoked or units of alcohol consumed |  |
| Element | Clinical self-management behaviours | Outcome measures for assessing patient or carer adherence to expected clinical self-management behaviours  Example: adherence to medications |  |
| Element | Psychological management behaviours | Outcome measures for assessing adherence to agreed-on psychological self-management behaviours  Example: management of stress level |  |
| Element | Social management behaviours | Outcome measures for assessing adherence to agreed-on self-management behaviours in social situations. |  |
| Element | Interactions and communication with healthcare and/or social care professionals | Outcome measures for assessing a patient’s capacity to interact and communicate with healthcare providers. |  |
| Subdomain | Clinical outcomes | This subdomain includes Clinical outcomes measures covering disease progression (clinical markers, symptoms), morbidity and/or complications, adverse events, and mortality. |  |
| Element | Disease progression (clinical markers, symptoms) | The worsening of disease over time. This concept is often used for chronic or incurable diseases where the stage of the disease is an important determinant of therapy and prognosis. | ^43^ |
| Element | Complications | Unfavourable progression or consequence of a long-term condition. |  |
| Element | Adverse events | Unexpected medical problem attributable to a self-management intervention. Adverse events can be mild, moderate, or severe, and may have other causes, such as medication or therapy. They are also known as *adverse effects*. |  |
| Element | Mortality | Mortality outcome measures include number of deaths by place, time, or cause observed before, during, or after the implementation of a self-management intervention. |  |
| Subdomain | Patient and informal caregivers’ quality of life | This subdomain includes outcome measures to assess overall and specific components of a petients or carer quality of life, including physical functioning, psychological functioning, social functioning, and burden of treatment. |  |
| Element | Overall quality of life | An individual’s perception of their position in life in the context of the culture and value systems in which they live and in relation to their goals, expectations, standards and concerns. | ^44^ |
| Element | Physical functioning | Involves basic actions and complex activities, activities considered essential for maintaining independence, and activities that are considered discretionary, i.e., that are not required for independent living but may nonetheless have an impact on quality of life. Commonly studied physical functions include activities such as walking and climbing stairs, but the role of environmental factors and behavioural strategies to compensate for reduced performance capacity or environmental barriers in characterizing physical functioning must also be taken into account. | ^45,46^ |
| Element | Psychological and emotional role functioning | Measures of anxiety, depression, etc. Also includes the extent to which physical health or emotional problems interfere with normal social activities with family, friends, neighbours, or groups. |  |
| Element | Social functioning | Ability to interact with one’s environment, fulfil one’s role in, for example, work and social situations, and maintain relationships with one’s partner and family. | ^47^ |
| Element | Sexual functioning | Ability to experience sexual pleasure and satisfaction when desired. Sexual pleasure can be experienced in many ways, including solo masturbation, oral sex, sensual massages, and a range of other possibilities. | ^48^ |
| Element | Burden of treatment | Burden of treatment is a dynamic process that varies over time in response to disease severity and control, treatment, and comorbidities. It can be both objective (e.g., in relation to the number of medications a patient needs to take or the time required to administer or monitor treatment) and subjective (e.g., a patient’s fears or concerns about treatment). Burden of treatment can be physical (e.g., side effects from medication, adverse events, injection pain), logistical (e.g., scheduling appointments or visits with healthcare professionals, organizing rehabilitation, arranging transport), technical (e.g., making lifestyle changes, taking medication, doing exercises), relational (e.g., securing support of family, friends, or health professionals, initiating interactions with possible caregivers), or temporal (e.g., time needed to travel or organize transport, receive treatment, learn about treatment, monitor treatment, manage side effects). Another type of burden is psychosocial time demands (dependency, fulfilment of roles, impact of treatment on significant others, treatment tasks that interfere with daily life, shame, stigma). | ^49^ |
| Subdomain | Care perception/Satisfaction | This subdomain includes outcome measures related to a patients’ experience of a self-management intervention. |  |
| Element | Overall satisfaction with self-management interventions | General feeling of satisfaction that patients and caregivers have with a self-management intervention in which they are participating. |  |
| Element | Perception of being well and sufficiently informed (quality of information provision) | Wide variety of perceptions, feelings, emotions and experience of patients and caregivers about the information they have received  Examples: information on their health, sources of healthcare information, treatment, and medication use and how they have received it. |  |
| Element | Perception of patient - provider relationship | Perceptions, feelings, and emotions that patients or caregivers have about communication, trust, and professional competence. |  |
| Element | Personalized care | Extent to which a patient’s needs and preferences are taken into account. |  |
| Subdomain | Healthcare use | This subdomain includes outcomes related to type and number of visits, hospital admissions and readmissions and visits to emergency care. |  |
| Element | Number of visits | Visits or contact with healthcare providers, including primary care, outpatient visits, etc. |  |
| Element | Hospital admissions and readmissions | Hospitalization and rehospitalization (usually defined as a new admission within 30 days of discharge). |  |
| Element | Emergency care | (Number of) emergency department visits (hospital). |  |
| Subdomain | Cost | This subdomain includes outcome measures related to costs at different levels, from an individual patient, to healthcare systemand society as a whole. |  |
| Element | Healthcare costs for patient | Direct medical costs incurred by a patient (out-of-pocket expenses) and/or an increase or reduction in insurance premiums.  Examples: Payment for a self-management app, reduction in an insurance premium for people who are physically active (measured by gym attendance). |  |
| Element | Healthcare costs | Costs to the healthcare system or savings achieved as a result of a self-management intervention. |  |
| Element | Direct non-medical costs | Costs not related to healthcare provision, such as transport for non-medical reasons, social care services (formal care), and carer time (informal care provided by non-professional caregivers, who are often relatives, but may also be friends or neighbours). |  |
| Element | Societal costs | Productivity losses due to absenteeism, disability pensions, premature deaths, etc. | ^50^ |
| Domain | Target population | This domain relates to the population targeted by an intervention defined by broad categories of disease-related, socioeconomic, and demographic characteristics, as they can all influence self-management. During the design of a self-management intervention, these characteristics could be taken into account to select tailored components. Interventions may or may not specifically target one of these population groups. |  |
| Subdomain | As defined by intervention recipient | This subdomain includes the broader categories of target population: patients, caregivers, and community. |  |
| Element | Patients | Target population formed by individual patients selected according to different characteristics, such as risk status, presence of comorbid conditions, or non-clinical characteristics. | ^51^ |
| Element | Informal or family caregivers | Target population formed by individuals who informally provide care to a person living with a chronic disease. They are normally relatives but may be friends or neighbours. |  |
| Subdomain | As defined by disease-related characteristics | This subdomain includes the main disease-related categories by which a population targeted by a self-management intervention could be defined: illness duration, severity, and comorbidity. |  |
| Element | Time since diagnosis | Target population defined by the time a patient has been living with a diagnosis. For example, an intervention could target recently diagnosed patients or patients who have been living with a diagnosis for years. |  |
| Element | Disease severity | Target population defined by the extent of organ system derangement or physiological decompensation. For example, interventions could target patients with disease of a certain severity, which can be identified by events such as hospitalization and acute episodes but also by more stable markers. |  |
| Element | Comorbidity and multi-morbidity | Target population defined by the concurrence of other diseases.  Example: interventions may target patients living with multiple diseases or a specific combination (e.g. Diabetes and depression). |  |
| Subdomain | As defined by socioeconomic or demographic characteristics | This subdomain includes the main non-disease related categories by which a population targeted by a self-management intervention could be defined: socioeconomic status, ethnic or cultural background, age, sex/gender, and living situation. |  |
| Element | Socioeconomic status | Target population defined by socioeconomic status, which could be measured as a composite metric based on an individual’s economic and social standing. Interventions might also define their target population by a specific metric of socioeconomic status, such as highest level of education attained, education of parents, current occupation, net income, household income, wealth (assets, capital), or other related variables. | ^52^ |
| Element | Cultural group | Target population defined by ethnic, religious, or other cultural backgrounds. These populations are usually smaller in numbers than other groups in a given health population. | ^53–57^ |
| Element | Health literacy | Target population defined by health literacy level. Health literacy is the degree to which individuals are able to obtain, process, and understand basic health information and services needed to make appropriate health decisions |  |
| Element | Digital literacy | Target population defined by digital literacy.  Digital literacy is the degree to which individuals are able to access, manage, understand, integrate, communicate, evaluate, and create information safely and appropriately through digital technologies. It includes competences that are variously referred to as computer literacy, ICT literacy, information literacy, and media literacy. | ^58,59^ |
| Element | Biological sex or gender | Target population defined by biological sex or socially constructed gender. Gender describes characteristics of women and men that are largely socially created, while sex encompasses characteristics that are biologically determined. However, these terms are often mistakenly used interchangeably in the scientific literature, health policy, and legislation. | ^60–63^ |
| Element | Age | Target population defined by age. |  |
| Element | Living situation | Target population defined by taking into consideration the form of the cohabitation unit (e.g., living alone, single parent). |  |

**References used for the definitions**

1. Improving Chronic Illness Care. Self‐management support. http://www.improvingchroniccare.org/index.php?p=SelfManagement_Support&s=39 Accessed April 15, 2021

2. Michie S, Ashford S, Sniehotta FF, Dombrowski SU, Bishop A, French DP. A refined taxonomy of behaviour change techniques to help people change their physical activity and healthy eating behaviours: the CALO-RE taxonomy. *Psychol Heal*. 2011;26(11):1479-1498. doi:10.1080/08870446.2010.540664

3. Parsons S, Bury M, Hurst P, Magee H, Taylor D. Self management support amongst older adults: the availability, impact and potential of locally based services and resources. . 2010;1‐249. https://njl-admin.nihr.ac.uk/document/download/2027589.

4. Graham L, Wright J, Walwyn R, et al. Measurement of adherence in a randomised controlled trial of a complex intervention: supported self-management for adults with learning disability and type 2 diabetes. *BMC Med Res Methodol*. 2016;16. doi:10.1186/s12874-016-0236-x

5. Pearce G, Parke HL, Pinnock H, et al. The PRISMS taxonomy of self-management support: derivation of a novel taxonomy and initial testing of its utility. *J Health Serv Res Policy*. 2015;0(0):1-10. doi:10.1177/1355819615602725

6. Coulter A, Parsons S, Askham J. Where are the patients in decision-making about their own care ? 2008:14. http://www.who.int/management/general/decisionmaking/WhereArePatientsinDecisionMaking.pdf.

7. EMPATHiE Consortium. Final Summary Report: EMPATHiE, Empowering Patients in the Management of Chronic Diseases. 2014. https://www.eu-patient.eu/Members/Weekly-Mailing/empathie-finalreport/

8. Dwarswaard J, Bakker EJM, van Staa A, Boeije HR. Self-management support from the perspective of patients with a chronic condition: A thematic synthesis of qualitative studies. *Heal Expect*. 2016;19(2):194-208. doi:10.1111/hex.12346

9. Michie S, Wood CE, Johnston M, Abraham C, Francis JJ, Hardeman W. Behaviour change techniques: the development and evaluation of a taxonomic method for reporting and describing behaviour change interventions (a suite of five studies involving consensus methods, randomised controlled trials and analysis of qualitative data). *Health Technol Assess*. 2015;19(99):1-188. doi:10.3310/hta19990

10. Street RL, Slee C, Kalauokalani DK, Dean DE, Tancredi DJ, Kravitz RL. Improving physician-patient communication about cancer pain with a tailored education-coaching intervention. *Patient Educ Couns*. 80:42-47. doi:10.1016/j.pec.2009.10.009

11. Lindner H, Menzies D, Kelly J, Taylor S, Shearer M. Coaching for behaviour change in chronic disease: A review of the literature and the implications for coaching as a self-management intervention. *Aust J Prim Health*. 2003;9(3):177. doi:10.1071/PY03044

12. Olszewska J. Rehabilitation for chronic obstructive pulmonary disease patients. *Polish Ann Med*. 2011;18(1):177-187. doi:10.1016/S1230-8013(11)70037-6

13. Regenstrief Institute. Encounter type / Patient encounter type - LOINCS data base. Logical Observation Identifiers Names and Codes (LOINC) Committee. https://r.details.loinc.org/AnswerList/LL3016-4.html. Published 2018. Accessed August 17, 2018.

14. eHealth Ontario. Health Care Encounters | eHealth Blueprint. https://www.ehealthblueprint.com/en/documentation/chapter/health-care-encounters. Published 2018. Accessed August 29, 2018.

15. Bärnighausen T. Population health intervention research: three important advancements. *Int J Public Health*. 2017;62(8):841-843. doi:10.1007/s00038-017-0985-2

16. Black DR, Cameron R. Self‐administered interventions: a health education strategy for improving population health. Heal Educ Res . 1997;12(4):531‐545.

17. The American Occupational Therapy Association. How Does Occupational Therapy Benefit Persons With Chronic Diseases? Chronic Disease Management Fact Sheet. doi:10.5014/ajot.2014.686S06

18. Tooley A. Importance of Self-Care in Social Work and Social Work Education. https://www.onlinemswprograms.com/in-focus/self-care-in-social-work-and-social-work-education.html. Published 2018. Accessed August 29, 2018.

19. Mental Health America. What is a Peer? | Mental Health America. http://www.mentalhealthamerica.net/conditions/what-peer. Published 2018. Accessed August 29, 2018.

20. Oxford Dictionaries. https://en.oxforddictionaries.com/definition/layman. Accessed August 29, 2018.

21. Committee on Assuring the Health of the Public in the 21st Century. The Future of the Public’s Health in the 21st Century. *Inst Med*. 2002. https://www.ncbi.nlm.nih.gov/books/NBK221245/. Accessed August 29, 2018.

22. McLeroy KR, Norton BL, Kegler MC, Burdine JN, Sumaya C V. Community-based interventions. *Am J Public Health*. 2003;93(4):529-533. http://www.ncbi.nlm.nih.gov/pubmed/12660190. Accessed May 18, 2018.

23. World Health Organization. WHO. *Adherence to Long-Term Therapies - Evidence for Action*.; 2003. http://apps.who.int/medicinedocs/en/d/Js4883e/6.html. Accessed August 29, 2018.

24. *People with Long-Term Conditions and Attitudes towards Physical Activity*.; 2016. https://richmondgroupofcharities.org.uk/sites/default/files/richmond_group_debrief_final_1.pdf. Accessed August 29, 2018.

25. Fusco D, Lattanzio F, Tosato M, et al. Development of CRIteria to assess appropriate medication use among elderly complex patients (CRIME) project: rationale and methodology. Drugs Aging. 2009;26(Suppl 1):3‐13.

26. Escola Galega de Saúde para Cidadáns. Uso adecuado de los medicamentos. https://escolasaude.sergas.es/Docs/EGSPC/pilula/uso_medicamentos/index.html#lc_es_p1. Published 2018. Accessed August 29, 2018.

27. Dobbels F, Van D‐L, Vanhaecke J, De GS. Growing pains: non‐adherence with the immunosuppressive regimen in adolescent transplant recipients. Pediatr Transplant. 2005 Jun;9(3):381‐390.

28. Jimmy B, Jose J. Patient medication adherence: measures in daily practice. Oman Med J. 2011 May;26(3):155‐159.

29. Quality Improvement for Institutions. *The Roles of Early Patient Recognition of Signs and Symptoms in Reducing Readmissions of HF and AMI Patients*. https://cvquality.acc.org/docs/default-source/h2h/4-symptom-managment-evidence-slides.pdf?sfvrsn=2ed58fbf_2. Accessed August 29, 2018.

30. Zambroski CH, Moser DK, Bhat G, Ziegler C. Impact of Symptom Prevalence and Symptom Burden on Quality of Life in Patients with Heart Failure. *Eur J Cardiovasc Nurs*. 2005;4(3):198-206. doi:10.1016/j.ejcnurse.2005.03.010

31. Kaye R, Crowley J. *Medical Device Use-Safety: Incorporating Human Factors Engineering into Risk Management*.; 2000. https://goo.gl/f9Kfdi. Accessed August 29, 2018.

32. Roditi D, Robinson ME. The role of psychological interventions in the management of patients with chronic pain. *Psychol Res Behav Manag*. 2011;4:41-49. doi:10.2147/PRBM.S15375

33. Skills you need. Helping You Develop Like Skills. Recognising and Managing Emotions | SkillsYouNeed. https://www.skillsyouneed.com/ps/managing-emotions.html. Accessed August 29, 2018.

34. National Institutes of Health U.S. National Library of Medicine. Consumer Health Informatics Research Resource - Sel-Efficacy. https://chirr.nlm.nih.gov/self-efficacy.php. Accessed August 29, 2018.

35. Lawrance L, McLeroy KR. Self-efficacy and Health Education. *J Sch Health*. 1986;56(8):317-321. doi:10.1111/j.1746-1561.1986.tb05761.x

36. Greene J, Hibbard JH, Sacks R, Overton V, Parrotta CD. When patient activation levels change, health outcomes and costs change,too. Health Aff (Millwood). 2015;34(3):431‐437.

37. Campbell ZC, Stevenson JK, McCaffery KJ, et al. Interventions for improving health literacy in people with chronic kidney disease. *Cochrane Database Syst Rev*. February 2016. doi:10.1002/14651858.CD012026

38. Blakemore A, Hann M, Howells K, et al. Patient activation in older people with long‐term conditions and multimorbidity: correlates and change in a cohort study in the United Kingdom. BMC Health Serv Res. 2016 Oct 18;16(1):582.

39. Hibbard JH, Stockard J, Mahoney ER, Tusler M. Development of the Patient Activation Measure (PAM): conceptualizing and measuring activation in patients and consumers. *Health Serv Res*. 2004;39(4 Pt 1):1005-1026. doi:10.1111/j.1475-6773.2004.00269.x

40. Hibbard JH, Mahoney ER, Stockard J, Tusler M. Development and Testing of a Short Form of the Patient Activation Measure. *Health Serv Res*. 2005;40(6p1):1918-1930. doi:10.1111/j.1475-6773.2005.00438.x

41. Heijmans M, Lemmens L, Otten W, Havers J. Zelfmanagement door mensen met chronische ziekten Kennissynthese van onderzoek en implementatie in Nederland. 2015. https://www.nivel.nl/sites/default/files/bestanden/Kennissynthese-Zelfmanagement.pdf. Accessed April 27, 2018.

42. Van Houtum L, Rijken M, Heijmans M, Groenewegen P. Patient-Perceived Self-Management Tasks and Support Needs of People with Chronic Illness: Generic or Disease Specific? 2014. https://www.ncbi.nlm.nih.gov/pubmed/25199663. Accessed April 26, 2018.

43. Medical subject Heading, Description of Data 2020. MeSH D018450.

44. Development of the World Health Organization WHOQOL-BREF quality of life assessment. The WHOQOL Group. *Psychol Med*. 1998;28(3):551-558.

45. Painter P, Stewart AL, Carey S. Physical functioning: definitions, measurement, and expectations. *Adv Ren Replace Ther*. 1999;6(2):110-123.

46. Tomey KM, Sowers MR. Assessment of physical functioning: a conceptual model encompassing environmental factors and individual compensation strategies. *Phys Ther*. 2009;89(7):705-714. doi:10.2522/ptj.20080213

47. Bosc M. Assessment of social functioning in depression. Compr Psychiatry. 2000 Jan-Feb;41(1):63‐69.

48. ASHA. American Sexual Health Association. Sexual Functioning |.

49. Sav A, King MA, Whitty JA, et al. Burden of treatment for chronic illness: A concept analysis and review of the literature. *Heal Expect*. 2015;18(3):312-324. doi:10.1111/hex.12046

50. Seidell JC. Societal and personal costs of obesity. Exp Clin Endocrinol Diabetes. 1998;106(Suppl 02):7‐9.51. Krumholz HM, Currie PM, Riegel B, et al. A taxonomy for disease management: A scientific statement from the American Heart Association Disease Management Taxonomy Writing Group. *Circulation*. 2006;114(13):1432-1445. doi:10.1161/CIRCULATIONAHA.106.177322

52. Socioeconomic Status - an overview | ScienceDirect Topics. https://www.sciencedirect.com/topics/medicine-and-dentistry/socioeconomic-status. Accessed May 18, 2018.

53. Collins-McNeil J, Edwards CL, Batch BC, Benbow D, McDougald CS, Sharpe D. A culturally targeted self-management program for African Americans with type 2 diabetes mellitus. *Can J Nurs Res*. 2012;44(4):126-141.

54. Council of Europe. *Cultural Diversity and Minorities T-Kit No.11 - MOSAIC - The Training Kit for Euro-Mediterranean Youth Work*.

55. Simpson GE, Yinger JM (John M. *Racial and Cultural Minorities an Analysis of Prejudice and Discrimination*. Harper; 1958.

56. Dobzhansky T. The science of man in the world crisis. Edited by Ralph Linton. Columbia University Press, New York, XIV + 532 pp., 1945. ($4.00). *Am J Phys Anthropol*. 1945;3(1):105-106. doi:10.1002/ajpa.1330030120

57. UNESCO. UNESCO Thesaurus: Cultural minorities. http://vocabularies.unesco.org/browser/thesaurus/en/page/?uri=http://vocabularies.unesco.org/thesaurus/concept7006. Published 2006. Accessed May 18, 2018.

58. Novilo Ortiz D. *Digital Health Literacy. World Health Organization. PAHO/WHO*.

59. Van der Vaart R, Drossaert C. Development of the digital health literacy instrument: measuring a broad spectrum of health 1.0 and health 2.0 skills. J Med Internet Res. 2017 Jan 24;19(1):e27.

60. World Health Organization. Genomic resource centre. WHO | Gender and Genetics. *WHO*. 2010.

61. Boonsatean W, Carlsson A, Dychawy Rosner I, Östman M. Sex‐related illness perception and self‐management of a Thai type 2 diabetes population: a cross‐sectional descriptive design. BMC Endocr Disord. 2018 Jan 30;18(1):5.

62. Cline RJ. The effects of biological sex and psychological gender on reported and behavioral intimacy and control of self‐disclosure. *Commun Q*. 1986;34(1):41-54. doi:10.1080/01463378609369619

63. Newman T. Sex and gender: What is the difference? Medical News Today.
